# Transgastric Synthetic Mesh Migration, 9 Years after Liver Resection

**DOI:** 10.1155/2014/412594

**Published:** 2014-04-15

**Authors:** Jae You, Neil Onizuka, Linda Wong

**Affiliations:** ^1^Department of Surgery, University of Hawaii John A. Burns School of Medicine, Queen's University Tower, 1356 Lusitana Street, Honolulu, HI 96813, USA; ^2^Department of Surgery, University of Hawaii John A. Burns School of Medicine, 550 South Beretania Street, Suite 403, Honolulu, HI 96813, USA

## Abstract

Complications of synthetic mesh have been described in various hernia procedures including migration and erosion, but no previous report mentions this complication after liver resection. This case describes a patient who had undergone a left hepatic resection with mesh pledgets sutured along the cut edge of the liver. He remained complication-free until nine years later when he presented with weight loss and early satiety, and endoscopy revealed mesh within the lumen of the stomach. While still attached to the liver, the mesh had eroded into the lumen of the stomach and he ultimately required surgery to remove this. The use of synthetic mesh in hepatectomies and other abdominal procedures may require further consideration by surgeons regarding its relatively unknown tendency for migration and erosion.

## 1. Introduction

The use of synthetic mesh in intra-abdominal procedures has become commonplace in surgical practice today. Typically mesh is utilized to reduce tension and reinforce repairs in abdominal wall and hiatal hernias. Reports have documented mesh migration and need for reoperation to retrieve the offending mesh and repair the adjacent organs. This report presents an unusual case of synthetic mesh used in a liver resection and its eventual migration and intraluminal erosion into the stomach.

## 2. Case Report

The patient is a 76-year-old male with a history of chronic hepatitis B with well-compensated cirrhosis. Nine years prior to admission, he underwent nonanatomic left lateral liver resection for a 2 cm well-differentiated hepatocellular carcinoma using a “sandwich technique.” This involved anchoring two Teflon felt pledgets to the resection margin anteriorly and posteriorly with running Ethibond (Ethicon Inc., Cincinnati, OH) polyester sutures. This method was chosen to expedite the procedure and minimize bleeding in an elderly, cirrhotic patient. He recovered uneventfully although a year later he required percutaneous radiofrequency ablation in the left medial segment for a 1.1 cm nonlocal recurrence of hepatocellular carcinoma.

**Figure 1:**
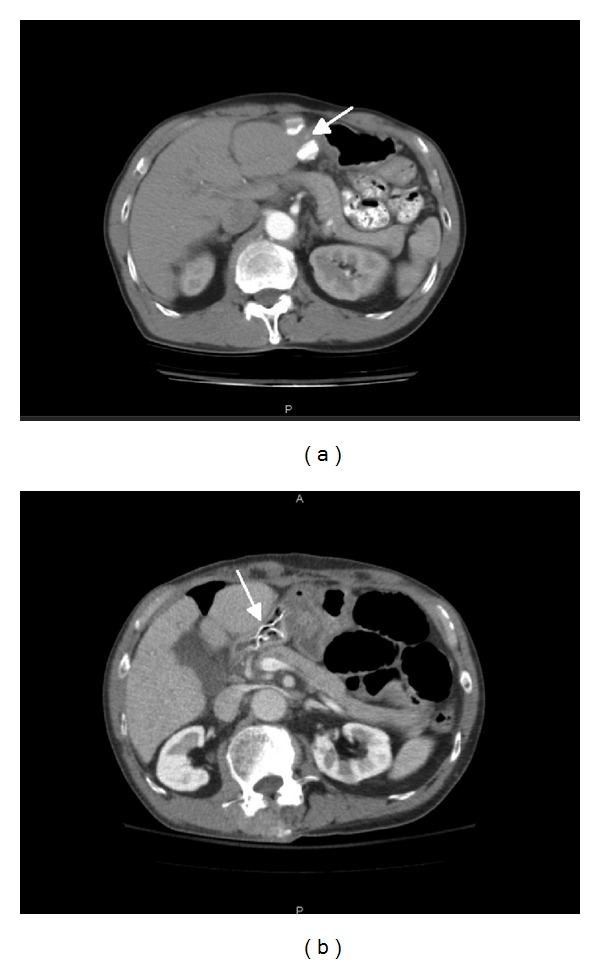
(a) Left, computed tomography scan 7 years after resection, showing the mesh (arrow) extraluminal to the stomach and duodenum. (b) Right, the mesh (arrow) migrated intraluminally 2 years later.

Nine years after resection, he complained of early satiety and a 10-pound weight loss. An esophagogastroduodenoscopy (EGD) revealed the Teflon felt within the lumen of the duodenal bulb with cleanly ulcerated mucosa. This had not been evident on EGD or CT scan (Figure 1(a)) four and two years prior, respectively. The mesh could not be removed endoscopically as it was quite adherent and there were concerns that the base was still sutured to a cirrhotic liver. However, the endoscope was able to move distally past the mesh indicating the absence of complete obstruction by the mesh.

Four hours after the EGD, the patient presented to the emergency department with severe abdominal pain and nausea. CT scan showed that the mesh was partially attached to the left medial segment of liver and passed transmurally into the lumen of the adjacent stomach and duodenum (Figure 1(b)). Much of the mesh appeared to line the anterior wall of the distal stomach and first portion of the duodenum. There was no evidence of perforation or abscess; however, there was also a distal small bowel obstruction unrelated to this mesh. Comparison with serial imaging for his hepatocellular cancer showed that the migration of this mesh likely occurred within the previous year. Shortly after admission, he developed transient bacteremia and hemodynamic instability requiring intensive care unit monitoring and resuscitation. Because of his cardiac arrhythmias, cirrhosis with borderline thrombocytopenia, and poor nutritional state, he was managed conservatively with nasogastric tube decompression, total parental nutrition, and broad-spectrum antibiotics.

His small bowel obstruction was not strangulated and resolved several days later. After 21 days, his nutritional parameters returned to normal and he had no evidence of sepsis or active infection. He then underwent an exploratory laparotomy and the lesser curvature and antrum of the stomach were densely adherent to the residual left medial segment of liver. There was concern of either causing bleeding at the cirrhotic liver edge and/or disrupting a significant portion of the anterior stomach, which might require an extensive gastrectomy. It was decided to approach this with a 4 cm gastrotomy on the posterior aspect of the stomach through which the mesh was visualized. The majority of the mesh was easily delivered from the first portion of the duodenum through the gastrotomy. A small point of fixation on the lesser curve towards the left lobe of the liver of the mesh was sharply divided and the base suture ligated. Two segments of mesh (12.5 × 0.8 cm) with attached sutures were then removed entirely and the posterior gastrotomy closed. A thick adhesive band in the small bowel of the right lower quadrant was identified as the likely site of the previous bowel obstruction though no dilated loops were seen. The patient tolerated the procedure without complication and was started on a diet on the fifth postoperative day. After receiving a cardiac pacemaker for his arrhythmias, he was discharged on postop day 10.

## 3. Literature Review and Discussion

Synthetic mesh is commonly utilized in a variety of hernia repairs for their superior tissue reinforcement and decreased recurrence rate when compared to nonmesh repairs. Complications related to mesh include postoperative hematomas, seromas, foreign body reaction, adhesions, infection, and mesh rejection. Erosions can occur with mesh in the intended position resulting in abscess, infection, and fistula [1]. Complete transmural migration of a mesh into an intraluminal position is a rare complication.

The exact pathophysiology of migration and erosion is not known. Predisposing factors include inadequate fixation, the presence of adhesions, and ongoing inflammation from previous surgeries. A plausible hypothesis is an inflammatory reaction to the sharp edges of the mesh, resulting in erosion of the parietal peritoneum and eventual luminal penetration into the intestine [1].

Mesh erosion and transmigration has been reported in various types of synthetic mesh including polypropylene plug mesh, polyester mesh, PTFE, and dual layered PTFE/ePTFE mesh. Each mesh type interacts with nearby tissues to varying degrees based on their innate biological properties. Polypropylene initiates an acute inflammatory response culminating in fibrosis, PTFE is associated with higher infection rates, and polyester tends to induce a chronic foreign body reaction [2]. The dual layered PTFE/ePTFE mesh combines a porous surface with an inert surface, which supports tissue growth on one side while allowing for mesh placement with minimal adhesions on the other [3]. However, no studies have been done comparing the incidence of transmigration and erosion in the different types of mesh.

The clinical presentation is varied and dependent on the site of migration. Migration of mesh into the bladder following inguinal hernia repair has been known to cause recurrent genitourinary tract infections. Other presentations of bladder migrations include hematuria, intravesical stone with mesh in its core, and colovesicular fistulas [2]. Presentation of mesh in the small bowel has been reported to cause obstruction and even perforation [1]. Mesh has been reported to be present intraluminally at different segments of colon, including the splenic flexure, cecum, and sigmoid [4]. Lange reported a case of a patient who presented with rectal bleeding due to sigmoid perforation following a mesh inguinal hernioplasty [5]. Mesh migration into at the esophagogastric junction may present as severe dysphagia due to the formation of an abscess with an inflammatory mass [2].

Management often requires an expeditious reoperation to remove the offending mesh. In cases presenting as intestinal obstruction due to transmigration of mesh, partial bowel resection is often required if the mesh does not spontaneously pass in the stool. Although transmigration is a rare complication of mesh use, it is dangerous and potentially fatal.

This case highlights a complication of synthetic mesh along the cut edge of liver and potentially other solid organs. While this practice is uncommon, surgeons may select this technique to minimize blood loss and assure quick hemostasis in patients who may be unable to tolerate prolonged hepatic surgery. This is the only report to describe such an event and additional cases may be necessary to establish a relationship between synthetic mesh on liver and potential complications. In general, mesh and other synthetic material are increasingly used for hernia repairs, antireflux surgery, and vascular surgery, many of which place synthetic material intraperitoneally and adjacent to luminal structures. Surgeons should inform patients of this potential complication before the antecedent procedure and be cautious when placing mesh adjacent to visceral structures. Prompt diagnosis of mesh migration can be difficult as we have illustrated that this phenomenon can occur many years after the initial surgery. Although mesh migration is a rare occurrence, surgeons should be aware of this potential complication and consider the possibility of mesh migration in patients with intestinal obstruction or unexplained abdominal sepsis and a history of synthetic mesh use.
